# Cytosolic Proteome Profiling of Aminoglycosides Resistant *Mycobacterium tuberculosis* Clinical Isolates Using MALDI-TOF/MS

**DOI:** 10.3389/fmicb.2016.01816

**Published:** 2016-11-15

**Authors:** Divakar Sharma, Manju Lata, Rananjay Singh, Nirmala Deo, Krishnamurthy Venkatesan, Deepa Bisht

**Affiliations:** Department of Biochemistry, National JALMA Institute for Leprosy and Other Mycobacterial DiseasesAgra, India

**Keywords:** amikacin, bioinformatic tools, kanamycin, cytosolic proteome, *Mycobacterium tuberculosis*, resistance

## Abstract

Emergence of extensively drug resistant tuberculosis (XDR-TB) is the consequence of the failure of second line TB treatment. Aminoglycosides are the important second line anti-TB drugs used to treat the multi drug resistant tuberculosis (MDR-TB). Main known mechanism of action of aminoglycosides is to inhibit the protein synthesis by inhibiting the normal functioning of ribosome. Primary target of aminoglycosides are the ribosomal RNA and its associated proteins. Various mechanisms have been proposed for aminoglycosides resistance but still some are unsolved. As proteins are involved in most of the biological processes, these act as a potential diagnostic markers and drug targets. In the present study we analyzed the purely cytosolic proteome of amikacin (AK) and kanamycin (KM) resistant *Mycobacterium tuberculosis* isolates by proteomic and bioinformatic approaches. Twenty protein spots were found to have over expressed in resistant isolates and were identified. Among these Rv3208A, Rv2623, Rv1360, Rv2140c, Rv1636, and Rv2185c are six proteins with unknown functions or undefined role. Docking results showed that AK and KM binds to the conserved domain (DUF, USP-A, Luciferase, PEBP and Polyketidecyclase/dehydrase domain) of these hypothetical proteins and over expression of these proteins might neutralize/modulate the effect of drug molecules. TBPred and GPS-PUP predicted cytoplasmic nature and potential pupylation sites within these identified proteins, respectively. String analysis also suggested that over expressed proteins along with their interactive partners might be involved in aminoglycosides resistance. Cumulative effect of these over expressed proteins could be involved in AK and KM resistance by mitigating the toxicity, repression of drug target and neutralizing affect. These findings need further exploitation for the expansion of newer therapeutics or diagnostic markers against AK and KM resistance so that an extreme condition like XDR-TB can be prevented.

## Introduction

Tuberculosis (TB) still remains one of the deadliest communicable diseases worldwide which is caused by *Mycobacterium tuberculosis*. WHO reported 9.0 million people developed TB and 1.5 million deaths including 3,60,000 people with HIV (WHO Report, 2015). Widespread development of multidrug-resistant tuberculosis (MDR-TB) has worsened the circumstances and aminoglycosides are used to treat them. Failure of aminoglycosides anti-TB treatment leads to resistance as well as emergence of extensively drug resistant tuberculosis (XDR-TB). Aminoglycosides, amikacin (AK) and kanamycin (KM) are important second line anti-mycobacterial drugs for MDR-TB patients. The principle targets of AK and KM resistance includes mutation in ribosomal protein/16S rRNA (Beauclerk and Cundliffe, 1987), enzymatic inactivation of drugs (Welch et al., 2005), decreased inner membrane transport and active efflux pumps (Magnet et al., 2001), cell wall impermeability (Nikaido, 2003), trapping of drug (Magnet et al., 2003). AK and KM resistance were contributed by rrs mutations in approximately 70% *M. tuberculosis;* however remaining 30% does not have these mutations and signifying the contribution of some other resistance mechanism (s). Advancement in proteomics has cleared the doubts to prove any complex phenotypes. As proteins marked most of the biological processes, these are attractive targets for developing new drugs and diagnostics against the resistance. Two-dimensional gel electrophoresis (2-DE) coupled with MALDI-TOF-MS have direct approaches for separation, identification, and characterization of proteins and its species (Kumar et al., 2013; Sharma et al., 2015b). Comparative proteomic studies addressing whole cell lysate and membrane and membrane associated proteins of aminoglycosides resistance isolates have been reported (Kumar et al., 2013; Sharma et al., 2015b). Recently we have reported involvement of ferritin (a cytoplasmic protein) in AK and KM resistance (Sharma et al., 2016). However, purely cytosolic expression proteome of aminoglycosides resistant *M. tuberculosis* isolates have not been explored. To address this issue, we analyzed purely isolated cytosolic proteins of AK and KM resistant *M. tuberculosis* by direct proteomic and bioinformatics approaches. Such information could be helpful for the development of newer diagnostics and drug targets against AK and KM drug resistance so that the situations like extensively drug resistance could be prevented.

## Materials and methods

### *M. tuberculosis* isolates collection and drug susceptibility testing

Four total susceptible (rifampicin, isoniazid, ethambutol, pyrazinamide, streptomycin, kanamycin, and amikacin) and four AK and KM resistant (sensitive to other first line and second line drugs) *M. tuberculosis* isolates were procured from Mycobacterial Repository Centre of National JALMA Institute for Leprosy and Other Mycobacterial Diseases, Agra, India. Drugs susceptibility testing (DST) for all the drugs was performed by LJ proportion (Canetti et al., 1969) and REMA methods (Palomino et al., 2002). Cultures were grown in Sauton's liquid medium at 37°C and harvested in late log phase (4 weeks) for proteomic analysis.

### Purely cytosolic proteins isolation and precipitation

Mycobacterial cell lysate was prepared with slight modifications (Brodie et al., 1979; Sharma and Bisht, 2016). Briefly, Cells were suspended in sonication buffer with 1% v/v Triton X-100 and then broken by intermittent sonication at 4°C for 20 min. Homogenate was centrifuged at 12,000 g for 20 min at 4°C. Resulting supernatants were ultracentrifuged at 150000 *g* for 90 min. to obtain the purely cytosolic supernatant and the pellet (cell membrane) was discarded. Cytosolic supernatant was precipitated using published protocol (Bisht et al., 2007). Protein concentrations were estimated by Bradford method (Bradford, 1976). Protein extractions were performed for three times in biological and technical replicas.

### Two dimensional gel electrophoresis

IEF and SDS-PAGE were carried out using the published protocol of “in gel rehydration” with slight modifications (Gorg et al., 2000; Sharma and Bisht, 2016). In brief, IPG strips of pH 4–7 and length 17 cm (Bio-Rad, Hercules, CA, USA) were rehydrated overnight at 20°C with 550 μg proteins. Strips were focused on an IEF unit (Bio-Rad) at 20°C. Proteins were separated in second dimension on 12% SDS-polyacrylamide gels in a vertical electrophoresis unit PROTEAN II XI (Bio-Rad). Gels were stained with Coomassie Brilliant Blue and analyzed using PDQuest Advanced software version 8.0.0 (BIORAD, Hercules, CA, USA). Protein spots which showed consistently increased intensities with more than 1.5 fold were selected for identification. Student t-test (inbuilt with software) was used for the statistical analysis by PDQuest Advanced software. The system picks up the spots with differential intensity of significant levels built in the system. Equal amount of protein was loaded in all gels and experiments were repeated in biological and technical replicates for at least three times.

### In gel digestion and mass spectrometry

In-gel digestion of proteins and MALDI-TOF/MS was carried out using published protocol (Shevchenko et al., 1996; Sharma et al., 2015b). In brief, mass spectra of digested proteins were acquired using Autoflex II TOF/TOF 50 (Bruker Daltonik GmbH, Leipzig, Germany). PMF were submitted to swissprot database for their identification taking taxonomy as *M. tuberculosis* complex. Peptide mass tolerance was set in range of 50–125 ppm (to acquire the best MASCOT score) with carbamidomethyl-cystein set as fixed modification. The oxidation of methionine was set as variable modification and one missed cleavage site was allowed.

### Bioinformatic analysis

Protein sequences of hypothetical proteins were retrieved from Tuberculist server http://tuberculist.epfl.ch/. Classes of proteins were predicted using TBpred server (Rashid et al., 2007). There probable functions and interacting partners were predicted using InterProScan, Molecular docking (**Table 3**), GPS-PUP (Quevillon et al., 2005; Schneidman-Duhovny et al., 2005; Andrusier et al., 2007; Mashiach et al., 2008; Liu et al., 2011) and STRING analysis (Search Tool for the Retrieval of Interacting Genes/Proteins, http://string.embl.de/) (Mawuenyega et al., 2005). STRING-10, server was used to predict the interacting partners of protein-protein interaction. STRING database uses a combination of prediction approaches and an integration of other information (neighborhood, transferred neighborhood, gene fusion, co-occurrence, co-expression, experiments, databases, text mining). Network was made at medium confidence level (0.400) allowing all active prediction methods.

## Results

**Figure 1:**
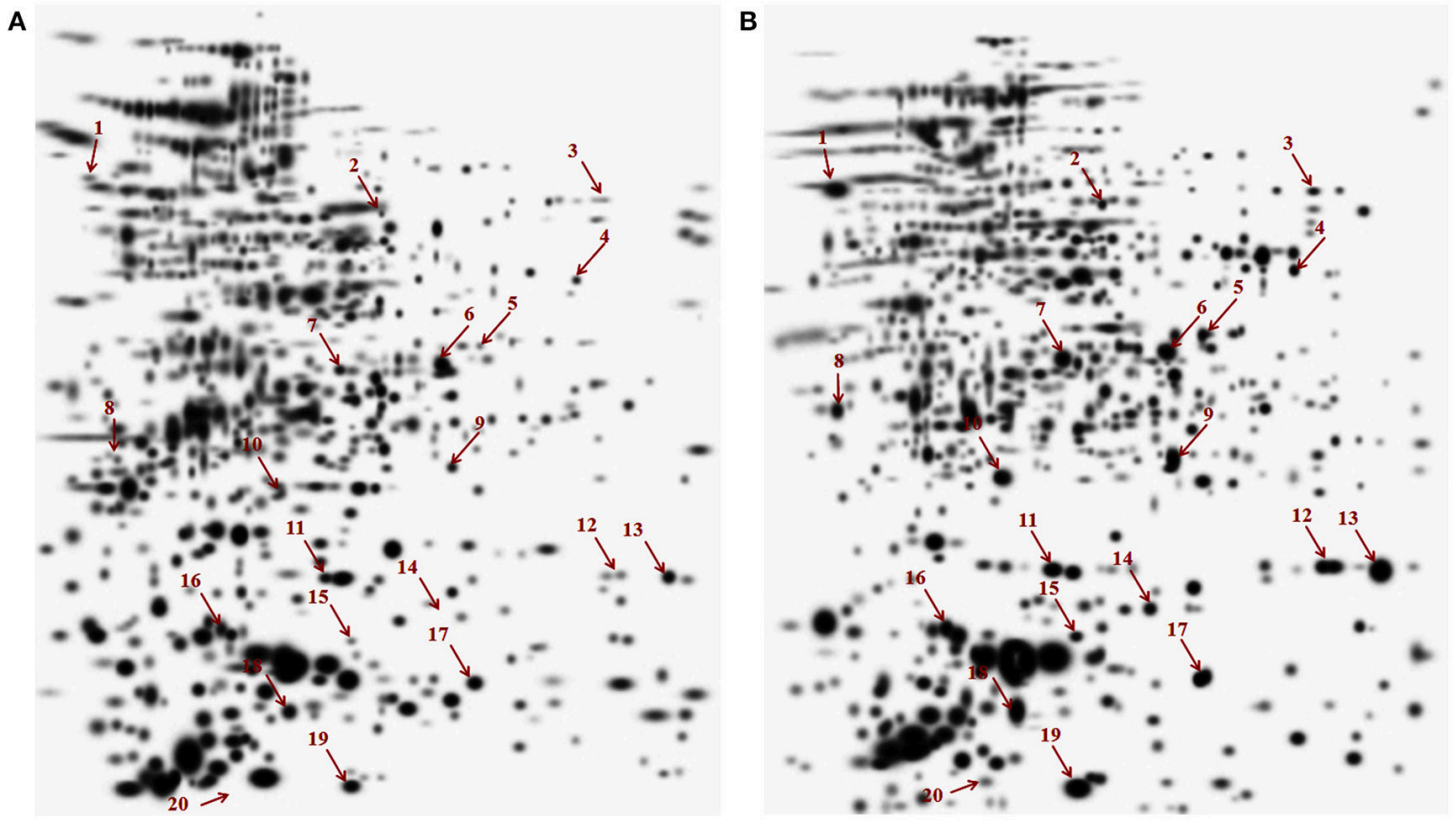
**Composite images of 2DE profile of cytosolic proteome of *M. tuberculosis isolates* (A) Total susceptible (B) AM and KM resistant isolates**.

In this study we have compared the cytosolic protein profiles of AK and KM resistant (lack *rrs* mutation) with total sensitive isolates. 2DE was run in triplicates for all isolates and composite images are shown in Figure 1. Comparison of 2D gels by PDQuest Advanced software revealed twenty protein spots which were consistently over expressed in resistant as compared to sensitive isolates (cut limit ≥ 1.5 fold changes in spot intensity). For statistical analysis inbuilt student *t*-test was used by PDQuest advanced software. Over expressed proteins spots were identified by MALDI-TOF-MS (Table 1). The identified proteins were Trigger factor (Rv2462c), 3-oxoacyl-[acyl-carrier-protein] synthase II (Rv2246), 3-ketoacyl-ACP reductase (Rv0242c), Transcriptional regulator MoxR1(Rv1479), Fructose-bisphosphate aldolase (Rv0363c), Universal stress protein (Rv2623), Uncharacterized Protein (Rv1360), Alkyl hydroperoxide reductase subunit C (Rv2428), Enoyl-coA hydratase (Rv0632c), Adenylate kinase (Rv0733), Single stranded DNA-binding protein (Rv0054), Peptidalprolyl isomerase (Rv0009), Beta carbonic anhydrase 1(Rv1284), UPF0098 protein (Rv2140c), Hypothetical protein (Rv2185c), Universal stress protein (Rv1636), Alpha-crystallin/HspX (Rv2031c), Probable cold shock protein A (Rv3648c), and Conserved Hypothetical protein (Rv3208A). However, two spots were identified as a protein of its species and therefore total 19 proteins were found. Among these Rv2623, Rv1360, Rv2140c, Rv2185c, Rv1636, and Rv3208A were proteins of unknown function. Out of nineteen, Rv0363c, Rv1360, Rv0733, and Rv1284 belonged to intermediary metabolism and respiration, Rv2623, Rv2428, and Rv1636, Rv2031c, and Rv3648c to virulence/detoxification/adaptation, Rv2462c to cell wall and cell processes, Rv2246, Rv0242c, and Rv0632c to lipid metabolism, Rv2140c, Rv2185c and Rv3208A to conserved hypothetical, Rv1479 to regulatory proteins and Rv0054 and Rv0009 to information pathway categories. The level of difference in protein spot intensity has been represented as densitometric ratio in Table 1. These proteins were also reported in cytosolic/whole cell lysate fraction of *M. tuberculosis* complex by various authors [23–26].

**Table 1:** **Details of proteins identified by mass spectrometry**.

**Spot No**.	**Accession number**	**Protein identified**	**MASCOT score**	**Nominal mass (Da)**	**pI**	**Sequence coverage %**	**ORF No**.	**Densitometric ratio of protein intensity between sensitive and resistant isolates**	Functional category^*^
D 1	P9WG55 (TIG_MYCTU)	Trigger factor	78	50586	4.47	33	Rv2462c	1: 3.80	2
D 2	P9WQD7 (FAB2_MYCTU)	3-oxoacyl-[acyl-carrier-protein] synthase II	133	44250	5.27	35	Rv2246	1: 2.50	7
D 3	O53665 (O53665_MYCTO)	3-ketoacyl-ACP reductase	198	46988	5.86	62	Rv0242c	1: 2.60	7
D 4	Q79FN7 (Q79FN7_MYCTU)	Transcriptional regulator MoxR1	117	40752	5.96	36	Rv1479	1: 2.20	4
D 5	P9WQA3 (ALF_MYCTU)	Fructose-bisphosphate aldolase	74	36522	5.49	29	Rv0363c	1: 3.50	1
D 6	P9WFD7 (Y2623_MYCTU)	Universal stress protein	70	31632	5.46	41	Rv2623	1: 1.83	5
D 7	P64831 (Y1360_MYCTU)	Uncharacterized Protein	68	37570	6.38	27	Rv1360	1: 2.50	1
D 8	P9WQB7 (AHPC_MYCTU)	Alkyl hydroperoxide reductase subunit C	142	21553	4.50	83	Rv2428	1: 2.90	5
D 9	P96907 (P96907_MYCTX)	Enoyl-coA hydratase	79	24281	5.52	57	Rv0632c	1: 3.98	7
D 10	P9WKF5 (KAD_MYCTU)	Adenylate kinase	168	20113	5.02	72	Rv0733	1: 3.60	1
D 11	P9WGD5 (SSB_MYCTU)	Single stranded DNA-binding protein	86	17343	5.12	40	Rv0054	1: 2.10	3
D 12	P9WHW3 (PPIA_MYCTU)	Peptidalprolyl isomerase	86	19271	5.80	54	Rv0009	1: 2.80	3
D 13	P9WHW3 (PPIA_MYCTU)	Peptidalprolyl isomerase	89	19271	5.80	54	Rv0009	1: 3.10	3
D 14	P9WPJ7 (MTCA1_MYCTU)	Beta carbonic anhydrase 1	117	18177	5.48	45%	Rv1284	1: 2.81	1
D 15	P9WFN1 (Y2140_MYCTU)	UPF0098 protein	137	18622	5.41	55	Rv2140c	1: 1.80	6,1
D16	O53519 (O53519_MYCTU)	Hypothetical protein	74	16314	4.75	45	Rv2185c	1: 1.90	6,7
D17	P9WFC9 (Y1636_MYCTU)	Universal stress protein	69	15303	5.51	44	Rv1636	1: 1.72	5
D 18	P9WMK1 (ACR_MYCTU)	Alpha-crystallin	63	16217	5.00	48	Rv2031c	1: 1.83	5
D 19	P9WP75 (CSPA_MYCTU)	Probable cold shock protein A	51	7366	5.17	83	Rv3648c	1: 2.60	5
D 20	Q6MWZ8 (Q6MWZ8_MYCTU)	Conserved Hypothetical protein	134	9884	4.86	90	Rv3208A	1: 1.90	6

*1, intermediary metabolism and respiration; 2, cell wall and cell processes; 3, information pathways; 4, regulatory proteins; 5, virulence, detoxification adaptation; 6, conserved hypothetical; 7, lipid metabolism*.

* *Note on functional category*.

### TBPred

TBPred analysis revealed that most of the identified proteins were found to be predicted in the class of cytoplasmic proteins (Table 2).

**Table 2:** **Prediction of classes of these identified proteins by TBpred server**.

**ORF No**.	**Score of amino acid composition based SVM approach**	**Score of dipeptide composition based SVM approach**	**Final predicted class of proteins**
Rv2462c	3.108	1.355	Cytoplasmic
Rv2246	0.927	0.949	Cytoplasmic
Rv0242c	0.704	−0.009	Integral MP/Cytoplasmic
Rv1479	2.402	0.057	Integral MP
Rv0363c	2.062	2.189	Cytoplasmic
Rv2623	1.061	0.549	Cytoplasmic/Integral MP
Rv1360	1.226	0.616	Cytoplasmic
Rv2428	0.417	0.460	Cytoplasmic
Rv0632c	0.676	0.482	Integral MP/Cytoplasmic
Rv0733	4.242	2.154	Cytoplasmic
Rv0054	0.319	−0.496	Attached to Membrane by Lipid Anchor/Secretory
Rv0009	0.553	0.223	Attached to Membrane by Lipid Anchor
Rv1284	1.762	1.515	Cytoplasmic
Rv2140c	0.110	0.081	Integral MP/Cytoplasmic
Rv2185c	3.738	2.543	Cytoplasmic
Rv1636	3.225	0.341	Cytoplasmic
Rv2031c	2.524	2.397	Cytoplasmic
Rv3648c	0.430	1.765	Integral MP/Cytoplasmic
Rv3208A	2.287	1.209	Cytoplasmic

### Interproscan analysis

InterProScan analysis of Rv3208A showed the presence of conserved domain (DUF3107) of protein of unknown function (#PF11305) from residues 1–73. Rv2623 (Universal stress protein) was found to be highly conserved in mycobacterial species and it appeared as hypothetical protein with unknown function. InterProScan analysis of Rv2623 showed the presence of two conserved USP-A domain with amino acid residues from 10 to 148 and 162–293 (#PF00582). Rv1360 showed conserved Luciferase like domain from residues 15–316 (#PF00296) and Rv2140c showed conserved Phosphatidylethanolamine binding protein (PEBP) domain (#SSF49777) from residues 17–174. Rv1636 showed the presence of conserved UspA domain with amino acid residues from 4 to 144 (#PF00582). Rv2185c showed the presence of Polyketide cyclase/dehydrase domain with amino acid residues from 4 to 143 (#PF10604).

### 3D modeling and docking

**Figure 2:**
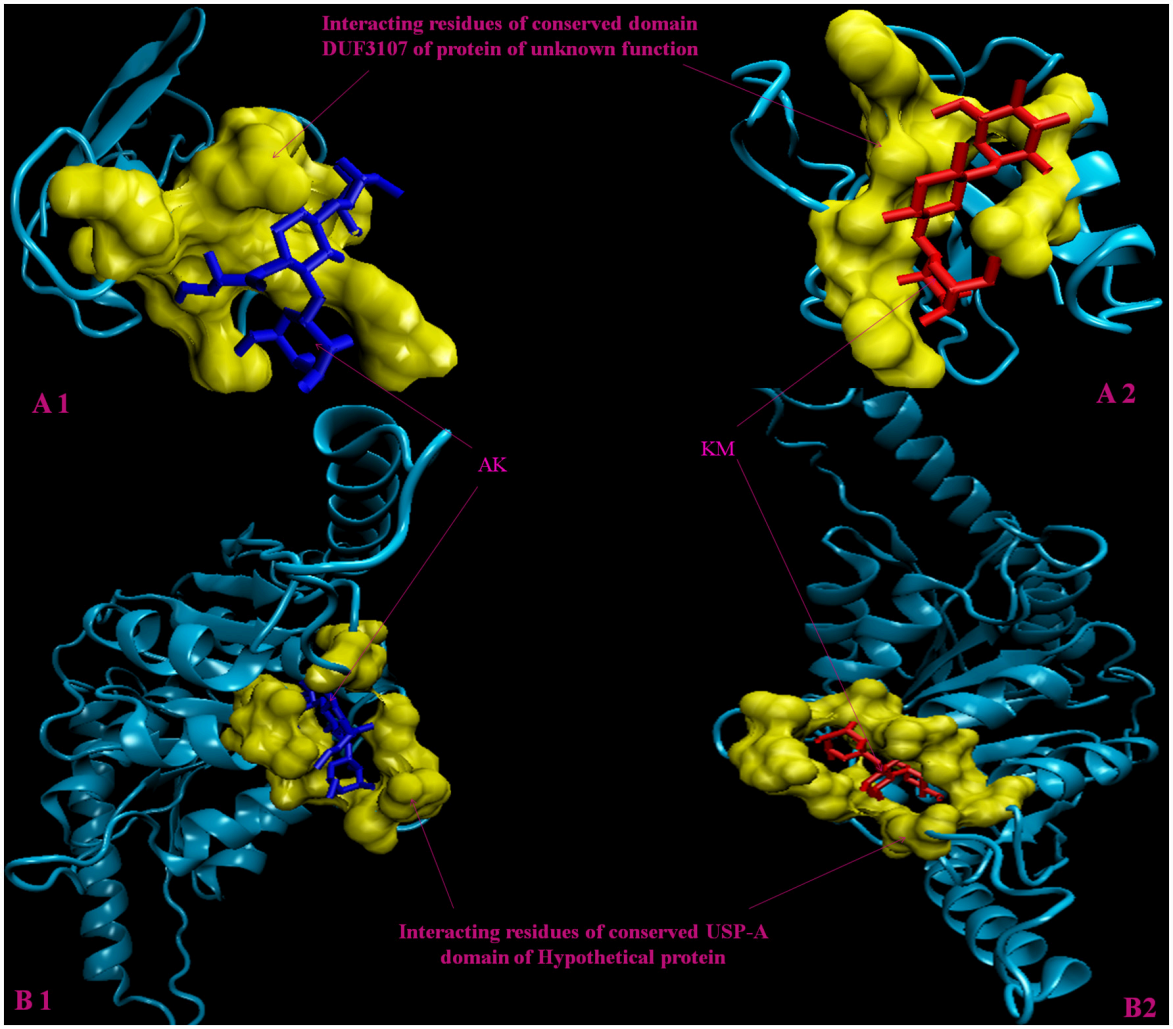
**3D model of hypothetical proteins showing docking with AK and KM. (A1,A2)** shows molecular docking of Rv3208A with AM (blue) and KM (red), respectively, yellow color shows interacting residues of DUF3107 domain. **(B1,B2)** shows molecular docking of Rv2623 with AM (blue) and KM (red), respectively, yellow color show interacting residues of USP-A domain.

**Figure 3:**
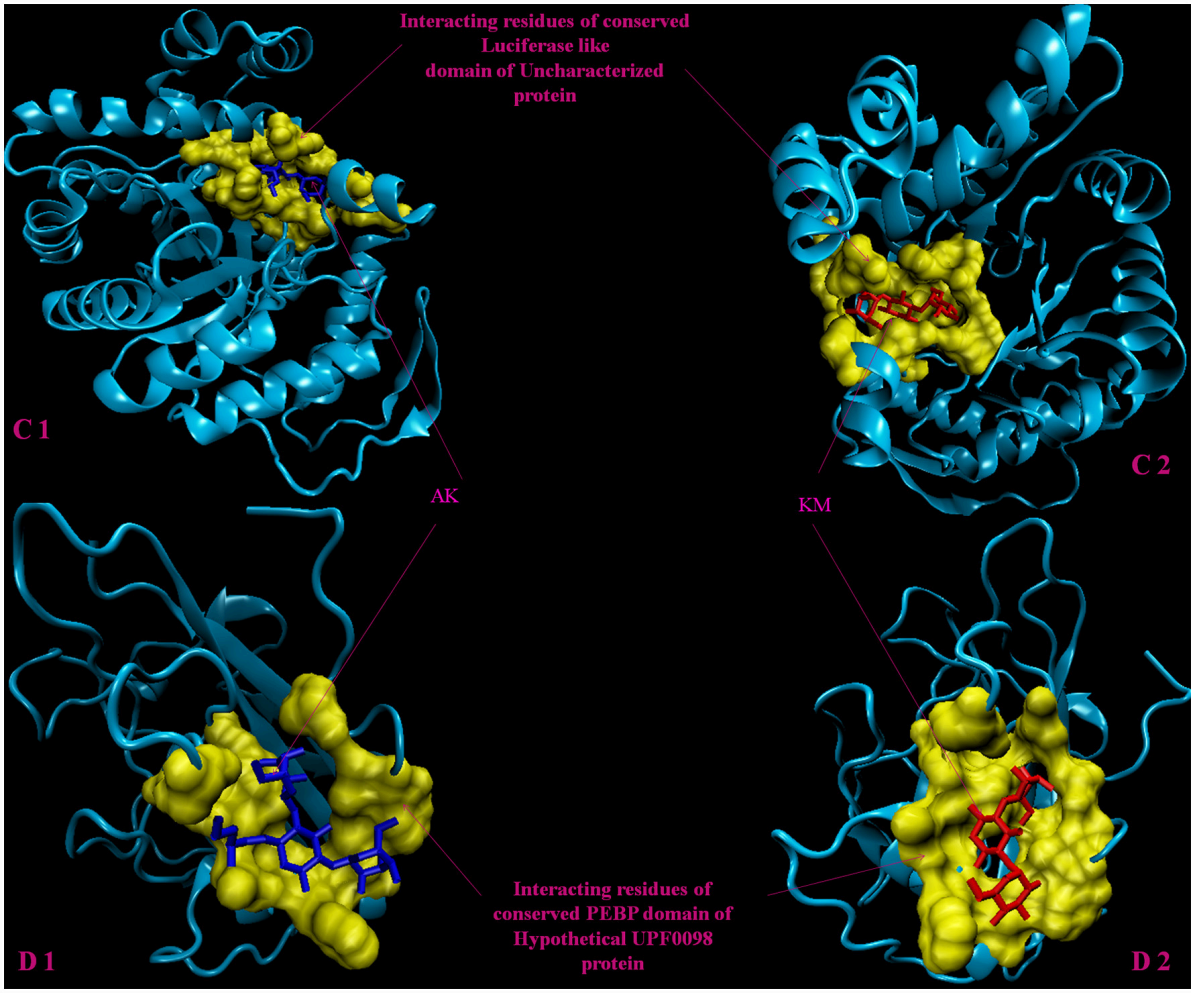
**3D model of hypothetical proteins showing docking with AK and KM. (C1,C2)** shows docking of Rv1360 with AM (blue) and KM (red), respectively, yellow color shows interacting residues of luciferase like domain. **(D1,D2)** shows docking of Rv2140c with AM (blue) and KM (red), respectively, yellow color shows interacting residues of conserved PEBP domain of hypothetical UPF0098 protein.

**Figure 4:**
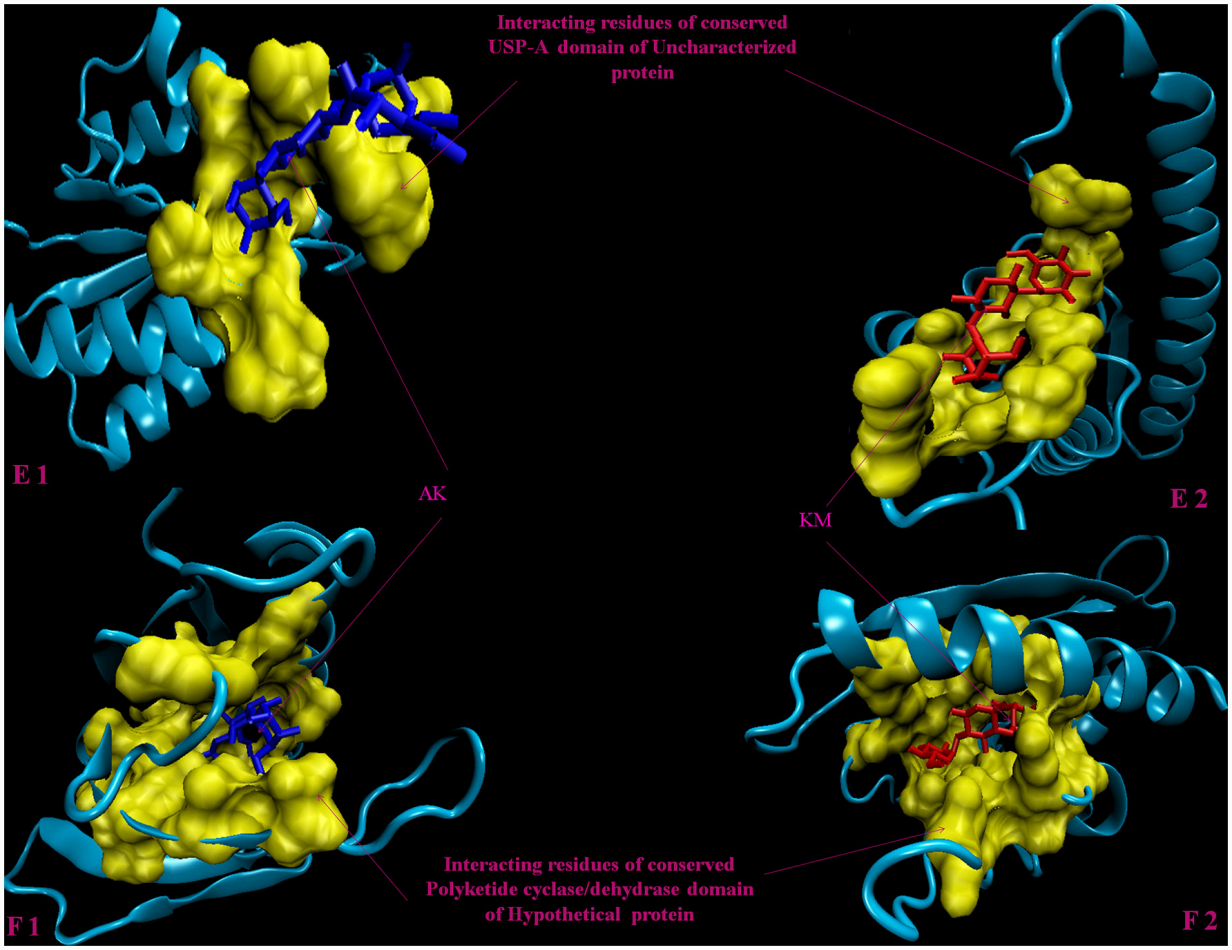
**3D model of hypothetical proteins showing docking with AK and KM. (E1,E2)** shows docking of Rv1636 with AM (blue) and KM (red), respectively, yellow color shows interacting residues of conserved USP-A domain, **(F1,F2)** shows docking of Rv2185c with AM (blue) and KM (red), respectively, yellow color shows interacting residues of conserved Polyketide cyclase/dehydrase domain of hypothetical protein.

Molecular docking analysis of selected 3D models (showing less than 2% discrepancy from Ramachandran plot) of hypothetical proteins was performed to find out their binding sites with AK and KM. Parameters used for selection of 3D models and molecular docking are represented in Table 3. Docking of Rv3208A (Figure 2) showed the interaction of AK and KM drugs to the conserved motif of domain of unknown function (DUF3107 domain). Docking with Rv2623 showed that both drugs bind to the residues of central cavity of USP-A domain of universal stress protein (Figure 2). For both drugs interacting residues were almost common, which suggests similar binding sites. Docking with Rv1360 (Figure 3) showed that both drugs bind to the interacting residues of conserved Luciferase like domain of the uncharacterized protein. Docking with Rv2140c showed that both drugs interacted with amino acids residues of conserved PEBP domain of hypothetical UPF0098 protein (Figure 3). Docking analysis of Rv1636 and Rv2185c (Figure 4) showed that AK and KM binds to conserved USP-A domain of uncharacterized protein and conserved Polyketide cyclase/dehydrase domain of hypothetical protein, respectively.

**Table 3:** **Modeling, docking parameters and analysis of interacting amino acids to drugs**.

**ORF No**.	**TM-score**	**RMSD value (Å)**	**Drug**	**Global energy**	**Attractive vander wall forces**	**Repulsive vander wall forces**	**ACE**	**Interacting amino acids**	**Remarks**
Rv3208A	0.37 ± 0.12	10.5 ± 4.6Å	AK	−24.82	18.86	11.20	−8.52	45–50, 69–72,74, 90	AK binds to conserved domain (DUF3107) of unknown function
Rv3208A	0.37 ± 0.12	10.5 ± 4.6Å	KM	−26.40	−16.16	3.07	−6.03	26, 30, 40, 42, 52, 53, 86, 88, 89	KM binds to conserved domain (DUF3107) of unknown function
Rv2623	0.95 ± 0.05	2.9 ± 2.1Å	AK	−55.14	−23.38	7.83	−17.58	167, 198, 261–263, 267–269, 272, 274–278	AK binds into central cavity of conserved USP-A domain
Rv2623	0.95 ± 0.05	2.9 ± 2.1Å	KM	−53.96	−23.57	2.53	−13.96	167, 198, 245, 262, 263, 265, 267–269, 272, 274–277	KM also binds into central cavity of conserved USP-A domain
Rv1360	0.88 ± 0.07	4.0 ± 2.7Å	AK	−51.96	−21.77	6.16	−15.20	99,110, 170–172, 174, 175, 188, 189, 196, 199, 252, 253, 255, 257, 258, 295	AK interact to conserved Luciferase like domain of uncharacterized protein
Rv1360	0.88 ± 0.07	4.0 ± 2.7Å	KM	−48.15	−21.32	3.57	−12.25	63, 64, 96, 98–00, 168–70, 174, 186–188, 223, 252, 253, 255	KM also interact to conserved Luciferase like domain of of uncharacterized protein
Rv2140c	0.97 ± 0.05	1.7 ± 1.4Å	AK	−41.65	−17.05	5.19	−15.42	7, 11, 12, 67, 75, 78, 153, 156, 157, 160	AK binds to PEBP domain of hypothetical UPF0098 protein
Rv2140c	0.97 ± 0.05	1.7 ± 1.4Å	KM	−42.41	−17.98	5.47	−14.14	7, 8, 11, 12, 67, 69, 75, 78, 153, 156, 157, 160, 166	KM binds to PEBP domain of hypothetical UPF0098 protein
Rv1636	0.80 ± 0.09	3.4 ± 2.4Å	AK	−26.95	−13.46	0.95	−9.50	114, 115, 121–125, 144–146	AK binds to binds to conserved USP-A domain of uncharacterized protein
Rv1636	0.80 ± 0.09	3.4 ± 2.4Å	KM	−33.03	−14.91	7.87	−11.76	41, 42, 44, 94, 115–118, 123, 126–128	KM binds to binds to conserved USP-A domain of uncharacterized protein
Rv2185c	0.82 ± 0.09	3.2 ± 2.3Å	AK	−45.57	−23.23	14.30	−13.91	28, 32, 35, 50, 54, 63, 65, 67, 69, 80, 88, 95, 108, 110, 123, 127, 130, 131, 134, 138	AK binds to conserved Polyketide cyclase/ dehydrase domain of hypothetical protein
Rv2185c	0.82 ± 0.09	3.2 ± 2.3Å	KM	−41.48	−17.75	2.17	−10.55	32, 34, 35, 54, 56, 63, 65, 80, 91, 95, 108, 110, 112, 123, 127, 130, 131, 134	KM binds to conserved Polyketide cyclase/ dehydrase domain of hypothetical protein

### Prediction of pupylation sites

Using the default threshold (medium) with cutoff 2.452, GPS-PUP predicted pupylation sites in 15 identified proteins which are tabulated in Table 4. Four proteins have not shown any pupylation sites with these default parameters.

**Table 4:** **Predicted/identified pupylation sites within identified proteins**.

**ORF No**.	**Position of lysine residue undergoes pupylation**	**Peptides**	**Score**	**Cut-off**
Rv2462c	2	^******^V**K**STVEQLS	2.614	2.452
	33	PDFQRAY**K**ELAKQVR	3.394	2.452
	274	SDQVRQA**K**RAQQAEQ	2.669	2.452
	439	DTSEFFG**K**RVSAGEA	2.677	2.452
Rv2246	54	TDAETTW**K**LLLDRQS	2.543	2.452
	164	VSPLTVQ**K**YMPNGAA	2.795	2.452
Rv0242c	100	ITEPAGL**K**GLHEFFT	2.591	2.452
	150	GFTRSLG**K**ELRRGAT	2.472	2.452
	168	VYLSPDA**K**PAATGLE	3.827	2.452
	381	LAPGLAA**K**GITINAV	3.323	2.452
Rv1479	71	MLVGLLS**K**GHVLLEG	2.646	2.452
Rv0363c	149	AIAQELL**K**AAAAAKI	3.669	2.452
	155	LKAAAAA**K**IILEIEI	3.016	2.452
	189	TSPEDFE**K**TIEALGA	3.165	2.452
	318	YDPRSYL**K**KAEASMS	2.52	2.452
Rv2623	35	ARDAELR**K**IPLTLVH	3.63	2.452
Rv2428	25	LIGGDLS**K**VDAKQPG	4.071	2.452
	70	TEIAAFS**K**LNDEFED	3.11	2.452
	192	LDAGELL**K**ASA^****^	6.024	2.452
Rv0733	23	QAVKLAE**K**LGIPQIS	4.26	2.452
	94	PRSVEQA**K**ALHEMLE	5.756	2.452
	181	RALRALGK^*******^	5.866	2.452
Rv0054	84	VIVSGRL**K**QRSFETR	2.866	2.452
	95	FETREGE**K**RTVIEVE	2.945	2.452
Rv0009	133	QFFITVG**K**TPHLNRR	2.646	2.452
	161	RVVEAIS**K**TATDGND	2.772	2.452
Rv2140c	133	QFFITVG**K**TPHLNRR	2.646	2.452
Rv2185c	89	LESSSLL**K**SLEGTYR	3.079	2.452
	136	RLIDGAL**K**DLKKRVE	3.236	2.452
	139	DGALKDL**K**KRVEG^**^	2.803	2.452
	140	GALKDL**K**KRVEG^***^	3.496	2.452
Rv2031c	64	LPGVDPD**K**DVDIMVR	2.669	2.452
	85	KAERTEQ**K**DFDGRSE	2.748	2.452
	114	GADEDDI**K**ATYDKGI	3.386	2.452
	119	DIKATYD**K**GILTVSV	2.913	2.452
	132	SVAVSEG**K**PTEKHIQ	3.433	2.452
Rv3648c	47	RTLEENQ**K**VEFEIGH	3.236	2.452
	57	FEIGHSP**K**GPQATGV	2.591	2.452
Rv3208A	85	AAAGSAG**K**VATSG^**^	4.126	2.452

*Bold letter “**K**” means it undergoes pupylation*.

^*^ It is the blank space where no amino acid occurs.

### String analysis

**Figure 5:**
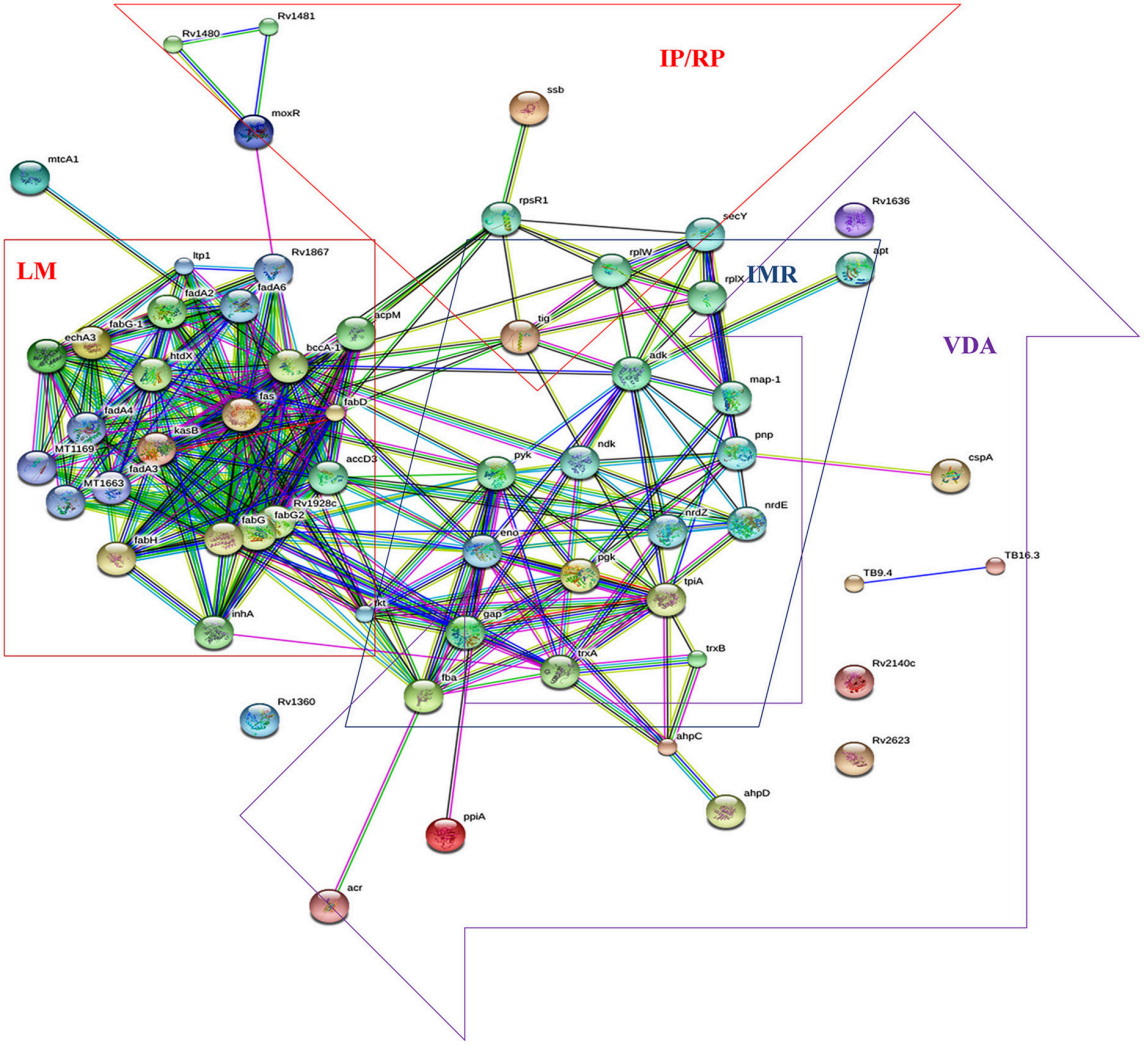
**STRING analysis reveals the interaction partners of the over expressed proteins which showed that over expressed proteins interacted to proteins of the similar functional categories except the hypothetical proteins**.

We analyzed the 19 over expressed proteins using STRING-10 with a medium confidence score threshold of 0.4 and build an interactome network (Figure 5) for these set of proteins to find out the protein-protein interaction (PPIs) and predict functional associations. We found that proteins involved in intermediary metabolism and respiration, lipid metabolism, information pathway/regulatory, and virulence, detoxification and adaptation category interacted with each other as well as their partners except the hypothetical proteins.

## Discussion

In this study we analyzed the comparative cytosolic proteome of AK and KM resistant isolates by 2DE-MALDI-TOF/MS and bioinformatic tools. Over expressed proteins in the resistant clinical isolates were identified, which may be used as diagnostic markers or drug targets for therapeutics. 2DE/MS has an advantage over the traditional methods (SDS-PAGE, chromatography and sequencing) as not only the identification of a large number of unknown proteins but also protein species separation. Several reports (whole cell lysates and membrane and membrane associated proteins) on aminoglycosides drug resistance and identification of diagnostics/drug targets employing proteomic and bioinformatic approaches exists (Sharma et al., 2010a, 2014, 2015a,b, 2016; de Souza et al., 2011; Kumar et al., 2013) which suggested that over expressed hypothetical proteins (Rv3867, Rv3224, Rv0148, Rv2744c), universal stress protein (Rv2005c) and some known proteins (involved in various pathways) might be involved in aminoglycosides resistance. However, to the best of our knowledge, no study on cytosolic proteome analysis with AK and KM resistant *M. tuberculosis* isolates has been reported.

### Repression of targets

Rv0363c (Fructose bisphosphate aldolase) is centrally involved in glycolysis, gluconeogenesis, and ATP synthesis. Mycobacteria reside in specialized niches and may require adaptations in the energy metabolism. It is essential for growth/pathogenesis of replicating and dormant *M. tuberculosis* (de la Paz Santangelo et al., 2011). Puckett et al. suggested that it regulates glycolytic and gluconeogenic carbon co-catabolism in *M. tuberculosis* (Puckett et al., 2014). Aminoglycosides resistant mycobacteria also behave like dormant bacilli due to repression of targets. Low translational state is an intrinsic defense mechanism of aminoglycosides resistant mycobacteria. Over expression of Rv0363c might maintain energy supply to resistant *M. tuberculosis*. Rv0733 (Adenylate kinase) is a small ubiquitous enzyme involved in ATP metabolism. Meena et al. suggested that Rv0733 has dual activity as nucleoside mono—and diphosphate kinase and could be implicated in the purine salvage pathway (Meena et al., 2003). Recently Lata et al. reported that Rv0733 is over expressed in ofloxacin mono resistant *M. tuberculosis* (Lata et al., 2015b). Over expression of Rv0733 maintains nucleotide supply to cells even in low translational state or dormant state and act as attractive targets for the development of new antimycobacterial agents. Rv1284 (β-carbonic anhydrase) catalyzes the reversible hydration of carbon dioxide to form bicarbonate and is also needed for fatty acid biosynthesis as well as pH homeostasis, therefore it is considered as essential for survival of *M.tuberculosis*. Rv1284 gene was highly over expressed under nutrients starved condition (Betts et al., 2002). Nienaber et al. reported Tyr120 of Rv1284 is critical residue for oxidative inactivation and its involvement in pH homeostasis/ redox-regulation (Nienaber et al., 2015). Over expression of Rv1284 might be involved in pathogenesis by maintaining the homeostasis/biosynthesis of fatty acids. Rv1479 (Transcriptional regulator MoxR1) is involved in regulatory function. Hu and Coates reported that its m-RNA was 4-fold over expressed in persisters as compared to stationary phase of mycobacteria (Hu and Coates, 2001). Recently Sharma et al. reported it's over expression in aminoglycosides resistant *M. tuberculosis* (Sharma et al., 2015b). Aminoglycosides resistant mycobacteria also behave like dormant bacilli due to repression of targets which is the mycobacterial intrinsic defense mechanism against resistance. Our study assumes that over expression of this protein regulate the transcription of mycobacteria even in low transcriptional state or dormant state. Rv0009 (Probable iron-regulated peptidyl prolyl cis–trans isomerase A) is probably involved in cis-trans isomerization of proline imidic peptide bonds in oligopeptides and accelerated protein folding (Henriksson et al., 2004). Recently Lata et al. reported the higher expression of Rv0009 in ofloxacin and moxifloxacin induced *M. tuberculosis* (Lata et al., 2015a). Over expression of Rv0009 might maintain cis-trans isomerization of amino acids and its supply to cells even in low translational state or dormant state.

We hypothesized that over expression of Rv0363c, Rv0733, Rv1284, Rv1479, and Rv0009 proteins might cumulatively overcome the effect of targets repression and contribute to aminoglycosides resistance.

### Mitigate the toxicity

Rv2462c (trigger factor, also acts as chaperone), is not only involved in protein export but also maintains the open conformation of newly synthesized protein. It was reported that its intensity is regulated by phosphate depletion (Rifat et al., 2009). Sharma et al. reported that it was over expressed in aminoglycosides resistant *M. tuberculosis* (Sharma et al., 2015b). Over expression of Rv2462c might mitigate the toxicity by maintaining the export of newly synthesized protein or truncated proteins in an open conformation. Rv2031c (Alpha-crystallin/HspX), protect the cells from different stimuli like stress, dormancy, drug and hypoxia by preventing protein aggregation (Sherman et al., 2001). In our previous studies, increased intensity of Rv2031c was reported in resistant *M. tuberculosis* (Kumar et al., 2013; Sharma et al., 2015b). Over expression of Rv2031c might mitigate the toxicity by preventing protein aggregation. Rv3648c (probable cold shock protein A) possibly involved in cold acclimation processes like dormancy, stress, drug and hypoxia (Hu et al., 1999). Rv3648c mRNA showed five to sevenfold increase in *Mycobacterium smegmatis* under cold-induction dormancy (Shires and Steyn, 2001). Over expression of Rv3648c might mitigate the aminoglycosides toxicity by maintaining the protein conformation. Rv2428 (Alkyl hydroperoxide reductase C/AhpC), a novel group of the peroxiredoxin family that reduces organic peroxide and hydrogen peroxides. Heym et al. reported that over expression of the AhpC involved in virulence and isoniazid resistance of *M. tuberculosis* (Heym et al., 1997). Lee et al. suggested that AhpC expression was induced under oxidative stress conditions and involved in *M. smegmatis* (Lee et al., 2014). Over expression of Rv2428 might mitigate the toxic effects of the aminoglycosides. Rv0054 (SS-DNA binding protein/SSB) involved to maintain DNA/RNA integrity in replication, recombination and repair phenomena. SSBs are also involved in modulating the activity of DNA polymerase, RNA polymerase and DNA helicase. Over expression of Rv0054 was found in drug induced study (Lata et al., 2015a). Over expression of Rv0054 might mitigate the toxicity/stress of drugs. Rv2246 (3-oxoacyl-[acyl-carrier protein] synthase 2) was involved in meromycolate elongation step of mycolic acid biosynthetic pathway (Singh et al., 2011) which provides a thick layer of lipid in the cell wall and protects *M. tuberculosis* from environmental and poisonous chemicals stress. Starck et al. reported that in *M. tuberculosis* Rv2246 was over expressed under anaerobic conditions (Starck et al., 2004). Over expression of Rv2246 might mitigate the toxicity of aminoglycosides. Rv0242c (Probable 3-oxoacyl-[acyl-carrier protein] reductase/FabG4) is involved in the first step of fatty acid biosynthesis pathway. Beste et al. suggested fabG4 to be essential for growth of *M. bovis BCG* on Roisin's medium (Beste et al., 2009). Sharma et al. suggested that Rv0242c might play a role in altering the drug permeability in resistant isolate by changing the fatty acid composition of the cell envelope (Sharma et al., 2010a). Over expression of Rv0242c might mitigate the toxicity of aminoglycosides. Rv0632c (Probable enoyl-CoA hydratase/EchA3) could possibly oxidize fatty acids. Maurya et al. reported that Rv0632c was overexpressed under anaerobic culture condition (Maurya et al., 2014). Recently Vargas-Romero et al. suggested that over expression of Rv0632c in hyper virulent *M. tuberculosis* CPT31 could facilitate mycobacterial infection and persistence (Vargas-Romero et al., 2016). Over expression of Rv0632c might mitigate the toxic effects of aminoglycosides.

We hypothesized that over expression of Rv2462c, Rv2031c, Rv3648c, Rv2428, Rv0054, Rv2246, Rv0242c, and Rv0632c proteins might cumulatively mitigate the toxicity and contribute to aminoglycosides resistance.

### Neutralizing effect

Rv2623 and Rv1636 are universal stress proteins with unknown function. These were over-expressed under nutrient/oxygen limitation and be involved in virulence/chronic infection (Stewart et al., 2001). Recently Lata et al. reported that Rv2623 and Rv1636 were over expressed in ofloxacin and moxifloxacin induced study (Lata et al., 2015a). In the present study we have observed that AK and KM interacted to the residues of conserved USP-A domains of Rv2623 and Rv1636, which might be altering the functions. It is suggested that over expression of these proteins might neutralize/compensate the effect of drugs. Rv1360 (probable oxidoreductase) is an uncharacterized protein, probably involved in cellular metabolism. Kruh et al. reported that Rv1360 was expressed in guinea pig model during early and chronic stages of disease (Kruh et al., 2010). The bacteria are thought to be in a state of reduced replication and metabolism as part of the chronic lung infection as well as in aminoglycosides resistance; therefore over expression of Rv1360 might maintain the replication and metabolism. Through *in-silico* approaches Raman and Chandra suggested that it is a potential drug target against tuberculosis (Raman and Chandra, 2008). In the present study we have observed that AK and KM interacted to the residues of conserved Luciferase like domain, which might be altering the functions. Rv2140c (UPF0098 protein/conserved hypothetical protein/TB18.6) is the protein of unknown function. It is reported that Rv2140c had a phosphatidyl ethanolamine-binding protein (Eulenburg et al., 2013). Recently Lata et al. reported that Rv2140c was over expressed in ofloxacin resistant *M. tuberculosis* (Lata et al., 2015b). In the present study we have observed that AK and KM interacted to the residues of conserved Phosphatidylethanolamine binding protein (PEBP) domain, which might be altering the functions. Rv2185c (Conserved hypothetical protein/TB16.3) is the protein of unknown function. Starck et al. reported that in *M. tuberculosis* Rv2185c was over expressed under anaerobic conditions (Starck et al., 2004). It is reported that over expression of Rv2185c induced IκBα degradation and nuclear translocation of NFκB (Zhang et al., 2015). In the present study we have observed that AK and KM interacted to the residues of conserved Polyketide cyclase/dehydrase domain, which might alter the function. Rv3208A (Conserved hypothetical protein/TB 9.4) is the protein of unknown function. Griffin et al. suggested that expression of Rv3208A is essential for *M. tuberculosis* growth and cholesterol catabolism (Griffin et al., 2011). In our study we have found that AK and KM interacted to the residues of conserved DUF3107 domain, which might alter the function.

We hypothesized that over expression of Rv2623, Rv1636, Rv1360, Rv2140c, Rv2185c, and Rv3208A hypothetical proteins might cumulatively neutralize/compensate the effect of drugs and contribute to aminoglycosides resistance.

Pupylation is a reversible PTM which is likely to have a regulatory role (Burns et al., 2010). Pupylation contributes to the virulence/survival strategy of *M. tuberculosis* in the host and makes the bacteria more resistant to drug and other stresses (Darwin et al., 2003). In our study, out of 19 over expressed proteins 15 showed the pupylation sites which suggested that over expression of these proteins via Pup-proteasome system (protein-protein interaction) might be involved in proteins turnover to overcome aminoglycosides stress. Interactome revealed that overexpressed proteins of lipid metabolism (LM), intermediary metabolism and respiration (IMR), information pathway/regulatory proteins (IP/RP), and virulence, detoxification and adaptation (VDA) category interacted to the other proteins which were involved in pathways of LM, IMR, IP/RP and VDA categories. We suggested that over expressed proteins along with their interactive partners of these various functional categories might cumulatively be involved in aminoglycosides resistance.

We assume that over expressed proteins might be contributing in AK and KM resistance and might act as diagnostic marker or potential targets for drug development against resistance.

## Conclusion

In a nutshell, in this report we focus on the purely cytosolic proteins of AK and KM resistant *M. tuberculosis* using proteomic and bioinformatic approach. Among the 19 over expressed proteins, 13 with their defined roles and six with unknown functions. TBpred predicted most of the proteins belong to cytoplasmic class. Molecular docking of these six hypothetical proteins (Rv2623, Rv1636, Rv1360, Rv2140c, Rv2185c, and Rv3208A) showed that AK and KM interacted to their conserved domains. GPS-PUP predicted presence of pupylation sites within 15 proteins. Interactome also suggested that over expressed proteins interacted with each other and their partners except the hypothetical proteins. It is depicting that over expression of these proteins might not only neutralize/ modulate the effect of drug molecules but also are involved in various mechanisms, such as in mitigating the toxicity, repression of drug target and protein turnover to overcome the AK and KM resistance. We assume that cumulative effect of these over expressed proteins might be responsible to AK and KM resistance. These findings need further exploitation for the development of newer therapeutic agents or molecular markers which can directly be targeted to a gene/protein responsible for resistance so that an extreme condition like XDR-TB can be prevented and which could ultimately lead to exploration of newer therapeutics.

## Author contributions

DS and DB design the concept of study, DS, ML, and RS involved in protein sample prepration and 2DGE, DS carried out the 2D-gel analysis, MALDI-TOF-MS and Bioinformatics analysis. DS, DB, ND, and KV finalized the manuscript.

## Funding

The research was financial supported by grant (No. 5/8/5/5/3/2011/-ECD-1) from ICMR, New Delhi. DS is ICMR-PDFs (ICMR, New Delhi).

### Conflict of interest statement

The authors declare that the research was conducted in the absence of any commercial or financial relationships that could be construed as a potential conflict of interest.
